# The world’s road to water scarcity: shortage and stress in the 20th century and pathways towards sustainability

**DOI:** 10.1038/srep38495

**Published:** 2016-12-09

**Authors:** M. Kummu, J. H. A. Guillaume, H. de Moel, S. Eisner, M. Flörke, M. Porkka, S. Siebert, T. I. E. Veldkamp, P. J. Ward

**Affiliations:** 1 Water & Development Research Group (WDRG), Aalto University, Espoo, Finland; 2 National Centre for Groundwater Research and Training & Integrated Catchment Assessment and Management Centre, The Fenner School of Environment and Society, The Australian National University, Australia; 3 Institute for Environmental Studies (IVM), Vrije Universiteit Amsterdam, Amsterdam, Netherlands; 4 Center for Environmental Systems Research (CESR), University of Kassel, Germany; 5 Institute of Crop Science and Resource Conservation (INRES), University of Bonn, Germany

## Abstract

Water scarcity is a rapidly growing concern around the globe, but little is known about how it has developed over time. This study provides a first assessment of continuous sub-national trajectories of blue water consumption, renewable freshwater availability, and water scarcity for the entire 20^th^ century. Water scarcity is analysed using the fundamental concepts of shortage (impacts due to low availability per capita) and stress (impacts due to high consumption relative to availability) which indicate difficulties in satisfying the needs of a population and overuse of resources respectively. While water consumption increased fourfold within the study period, the population under water scarcity increased from 0.24 billion (14% of global population) in the 1900s to 3.8 billion (58%) in the 2000s. Nearly all sub-national trajectories show an increasing trend in water scarcity. The concept of scarcity trajectory archetypes and shapes is introduced to characterize the historical development of water scarcity and suggest measures for alleviating water scarcity and increasing sustainability. Linking the scarcity trajectories to other datasets may help further deepen understanding of how trajectories relate to historical and future drivers, and hence help tackle these evolving challenges.

The overexploitation of freshwater resources threatens food security and the overall wellbeing of humankind in many parts of the world^1^. The maximum global potential for consumptive freshwater use (i.e. freshwater planetary boundary)^2^,^3^ is approaching rapidly^4^, regardless of the estimate used. Due to increasing population pressure, changing water consumption behaviour, and climate change, the challenge of keeping water consumption at sustainable levels is projected to become even more difficult in the near future^5^,^6^.

Although many studies have increased the understanding of current blue water scarcity^7^,^8^,^9^,^10^,^11^,^12^,^13^,^14^, and how this may increase in the future^5^,^6^,^15^, the historical development of water scarcity is less well understood^10^. Trajectories of these past changes at the global scale could be used to identify patterns of change, to provide a basis for addressing future challenges, and to highlight the similarities and differences in water scarcity problems that humanity shares around the world. This requires crossing scales, performing analyses globally, but at a sub-national resolution. Identifying recurring patterns of change can further provide evidence of key drivers of scarcity and thus help to recognise types of problems and solutions. Understanding what has occurred previously can thus help us to avoid repeating mistakes and to build on past successes.

Like other forms of scarcity, physical blue water scarcity can be fundamentally divided into two aspects: shortage and stress. Water *shortage* refers to the impact of low water availability per person. In “crowded” conditions, when a large population has to depend on limited resources, the capacity of the resource might become insufficient to satisfy otherwise small marginal demands, such as dilution of pollutants in a water body, and competition may result in disputes^16^. Given a resource and per capita requirements, water shortage can therefore be seen as population-driven scarcity. Water *stress* refers to the impact of high water use (either withdrawals or consumption) relative to water availability. Use of a large portion of a resource^1^,^13^ might lead to difficulties in accessing the resource, including side effects^16^, e.g. social and environmental impacts. Stress can be seen as demand-driven scarcity, potentially occurring even if the population is not large enough to cause shortage.

These two aspects have commonly been assessed in isolation from each other^7^,^10^, despite being combined in the seminal work on water scarcity by Falkenmark^1^,^16^,^17^, as well as some later works^15^,^18^. Indeed, the indicators of water shortage and stress are fundamentally related through per capita water use, and therefore provide a more complete picture when used together:


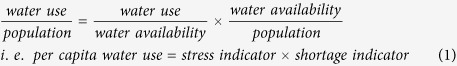


There are, however, multiple ways each of the terms can be defined, yielding different families of indicators for shortage and stress. For example, *use* can refer to consumption or withdrawals. *Availability* might refer to water from different sources, of different quality, or at decadal, annual or seasonal time scales. The *population* in question might be that which is dependent on a resource, which is physically located within a region, or only that which has access to the resource.

Given the complexity of the impacts, these are clearly crude indicators of actual impacts involved in stress and shortage. There is substantial uncertainty in determining at what value of the stress and shortage indicators, stress and shortage impacts actually occur. Even when justified thresholds are selected, the value of the indicator is typically also reported, so that the reader can form their own opinion of whether stress and shortage have really occurred.

Despite their high level of abstraction, and the multiple ways in which they can be used, the concepts of shortage and stress and their defining indicators are central to understanding the development of scarcity over time. Therefore, they provide an obvious first step in analysing trajectories of past changes.

This paper first explores how water consumption has evolved globally over the entire 20^th^ century. The analysis uses recently released spatially explicit data for the entire past century on socio-economic development^19^ and irrigation^20^, which allow us to assess past water consumption trends in greater detail, using the WaterGAP2 hydrological and water use models^19^,^21^ (see Methods). This evolution is put into context by assessment of water scarcity based on the concepts of shortage, stress and per-capita consumption, structured graphically using a Falkenmark matrix^1^,^16^,^17^. Archetypes and shapes of the trajectories are introduced as new concepts to characterize the historical development of water scarcity in regions, and hence to assess the effectiveness of potential alleviation strategies and define pathways towards sustainability.

The version of the shortage and stress indicators we use consider decadal scale water availability and consumption at sub-national scales. They therefore capture the effect of long term sub-national water scarcity, but not the seasonal variation in demand and supply, inter-annual variability or sub-regional variation. We focus on physical blue water scarcity, meaning that issues of access are omitted, and emphasis is on water in lakes, rivers and renewable groundwater rather than “green” water, soil water from precipitation directly used by plants, or non-renewable fossil groundwater. Moderate (high) shortage is deemed to occur when total water availability drops below a requirement of 1700 m^3^ cap^−1^ yr^−1^, (1000 m^3^ cap^−1^ yr^−1^)^1^,^7^. Moderate (high) stress is deemed to occur when more than 20% (40%) of available water is consumed^1^. The stress threshold was originally applied to water withdrawals but is used here for water consumption to account for substantial return flows that are still available for downstream users^22^,^23^. The focus on consumption also means that water degradation caused by return flows is not considered as part of stress, though it is still (indirectly) captured through population-driven pollutant load as part of shortage.

## Results

This study’s findings show a nearly 16-time increase in population under water scarcity since the 1900s although total population increased only 4-fold over the same time period. Per capita water consumption only shows a slight and irregular increase over the past century, while the expansion of water scarcity is predominantly explained by the effects of spatial distribution of population growth relative to water resources.

### Water consumption

**Figure 1:**
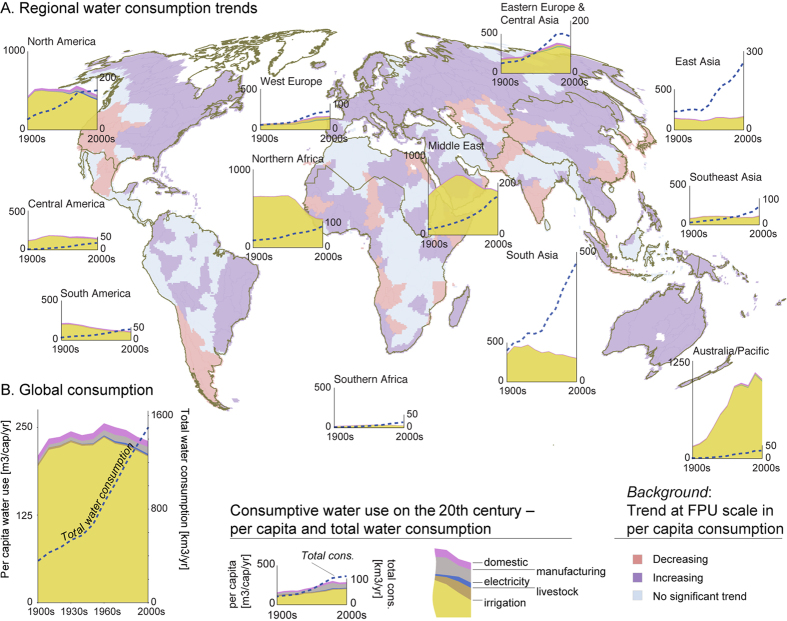
Regional (**A**) and global (**B**) consumptive water use trends over the 20^th^ century. The filled area represents per capita water consumption trends while the dashed line represents the total water consumption trends. The per capita consumption is divided into different water use sectors. The trend in per capita consumption at the FPU scale is shown as a background. [Adobe Illustrator CS5, ArcGIS 9.2 and Matlab 2015b softwares were used to create the figure; http://www.adobe.com/products/illustrator.html, http://www.esri.com, and http://www.mathworks.com].

The global population has almost quadrupled over the past hundred years, and it reached 6.5 billion in the last time step of the study period, i.e. the 2000s (given decadal results are averages over specified decades, in this case 2001–2010)^24^. Over the same period, annual *consumptive blue water use* per capita (see Methods for details) increased only from 209 m^3^ cap^−1^ yr^−1^ in the 1900s (i.e., 1901–1910) to 230 m^3^ cap^−1^ yr^−1^ in the 2000s, with some variation between decades and a maximum of 256 m^3^ cap^−1^ yr^−1^ in the 1960s (Fig. 1B). The increases in population and per capita water consumption resulted in a total water consumption increase from 358 km^3^ yr^−1^ in the 1900s to 1500 km^3^ yr^−1^ in the 2000s (Fig. 1B).

The trends of water consumption over the 20^th^ century were not, however, similar across the globe (Fig. 1A). The consumption per capita seems to have remained rather stable in many regions, such as Southern Africa and South America, but declined in the Middle East (since the 1950s), Northern Africa and South Asia. However, per capita consumption increased rapidly in Australia-Pacific, being over 6-fold greater in the 2000s compared to the 1900s. Increases were also found in Eastern Europe & Central Asia (until the 1990s) and Western Europe, although less rapid.

At the FPU (i.e., food production unit; see Methods) scale, this dataset shows that trends in per capita water consumption also varied significantly within the regions (Fig. 1A). A good example is North America, where the west coast experienced a decreasing trend while on the east coast, water consumption per capita increased. Of the world population, 46%, 25% and 29% live in FPUs where per capita consumption respectively increased, decreased, or showed no statistically significant trend over time (two-sided *p*-value > 0.05 with the Mann-Kendall test).

Although the trend in per capita water consumption varied between regions, total water consumption increased in all regions due to increased population except in Eastern Europe and Central Asia, where the total consumption decreased slightly (~7%) since the collapse of the Soviet Union in 1990 (Fig. 1A). Growth was greatest in Australia-Pacific (30-fold increase) followed by Central America, Southern Africa, and Southeast Asia (approximately eight-fold). In a number of regions, consumption increased 3–4 fold, with the lowest increase in Northern Africa with about a three-fold increase.

Globally, irrigation was by far the largest water consumer over the entire study period, with a share ranging over time between 90–94% of global water consumption (Supplementary Fig. 1B). It had the largest share in South Asia (96–98%) due to extensive rice cultivation, and in the Middle East (97–99%) due to arid conditions^20^. In Western Europe, the irrigation share of total water consumption was lowest (62–74%), as it includes areas where irrigation is not extensively practiced for food production. Moreover, the economy is more industrialised than, for example, in Asia. Globally, the second largest sector until the 1990s was domestic water consumption. However, this was surpassed by industrial water consumption in the final time step (2000s; domestic 3.7%, industrial 4.3%). A second notable global trend is the emergence of water consumption due to thermal electricity production (~1% share). Regionally, results show larger changes in the shares of different sectors, though the real-world significance of the changes is difficult to judge. In some areas (e.g. Western Europe, Australia/Pacific), the proportion of water consumption used for irrigation has increased and the proportion for domestic consumption has decreased. The opposite has occurred in other areas (e.g. North America, Supplementary Fig. 1A).

### Global and regional water scarcity

**Figure 2:**
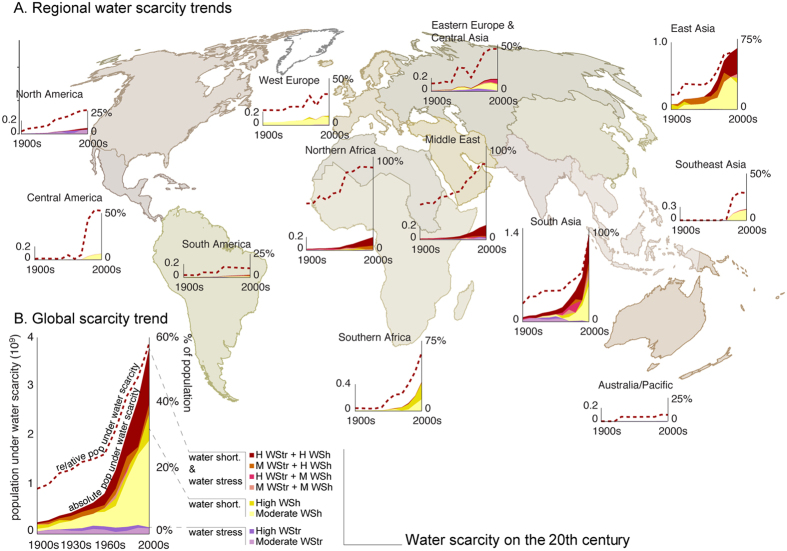
Regional (**A**) and global (**B**) water scarcity trajectories. Filled graphs represent the absolute population under water scarcity (in billions) while dashed lines represent the population relative to total regional population. M WStr refers to moderate water stress, H WStr to high water stress, M WSh to moderate water shortage, and H WSh to high water shortage. See definitions of these different water scarcity dimensions, and their combinations, in Table 1 and Fig. 4A. [Adobe Illustrator CS5, ArcGIS 9.2 and Matlab 2015b softwares were used to create the figure; http://www.adobe.com/products/illustrator.html, http://www.esri.com, and http://www.mathworks.com].

**Figure 3:**
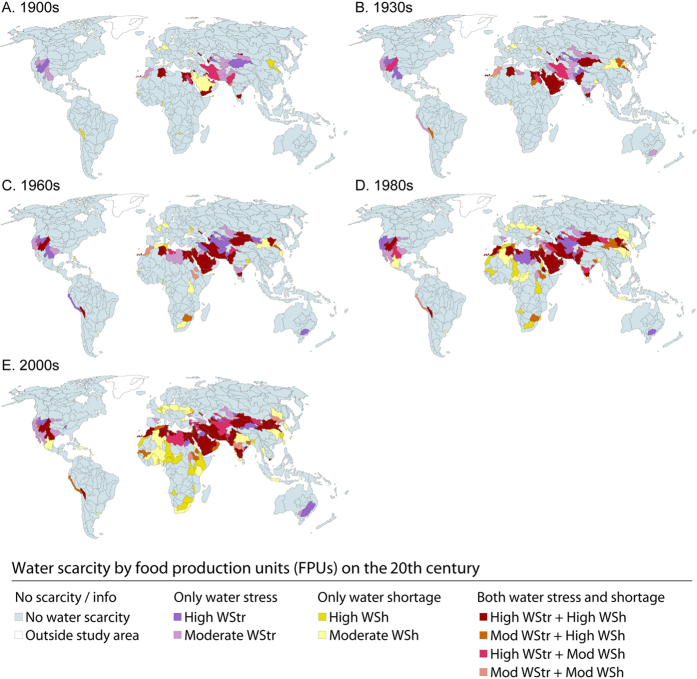
Mapped water scarcity categories for years 1905 (**A**), 1935 (**B**), 1965 (**C**), 1985 (**D**), and 2005 (**E**). The definition for each scarcity category is given in Table 1 and Fig. 4A. [Adobe Illustrator CS5 and ArcGIS 9.2 softwares were used to create the figure; http://www.adobe.com/products/illustrator.html, http://www.esri.com].

Despite only small variations in per capita water consumption over time (Fig. 1A), rapidly expanding local populations and increases in total water consumption resulted in a nearly 16-fold overall increase in the population under water scarcity within the 20^th^ century (Figs 2 and 3). Whilst in the 1900s just over 200 million people (14% of global population) lived in areas under some degree of water scarcity, this number increased to over two billion by the 1980s (42%), and reached 3.8 billion people (58%) by the 2000s (Table 1; Fig. 2B).

In the 2000s, roughly half of the people under water scarcity suffered either moderate water shortage or moderate water stress (Table 1), while the other half lived in areas facing both water stress and water shortage. Of these, 1.1 billion people (17% of global population) lived in areas facing both high water shortage and high water stress (Table 1; Fig. 2B). Most of these people lived in South and East Asia, North Africa and Middle East (Fig. 2A), with 61–89% of the population under water scarcity. The regions with the lowest proportion of population under water scarcity were Australia-Pacific, South America, North America, and Southeast Asia (7–29%, Fig. 2A). Around a half of the population under water scarcity in the 2000s suffered water shortage alone, without water stress (Table 1; Fig. 2B). These areas are located in Sub-Saharan Africa, Central America, Europe, and South and East Asia (Figs 2A and 3E). A small part of the population (2%) suffered water stress alone (Table 1), occurring mostly in North America, Middle East, and Australia (Fig. 3E).

A global water scarcity trend-plot (Fig. 2B) reveals that the population under water shortage, or a combination of high water stress and water shortage, has increased rapidly since the 1960s, while water stress alone has remained rather low over the entire study period. There are, however, differences in regional trajectories (Fig. 2A), indicating that, for example, in the Middle East, Northern Africa and North America, scarcity has developed gradually over the whole study period while in many other regions (e.g. Central America, Southern Africa, South Asia, Southeast Asia, and East Asia) there has been a steep increase in scarcity trend since the 1960s.

**Figure 4:**
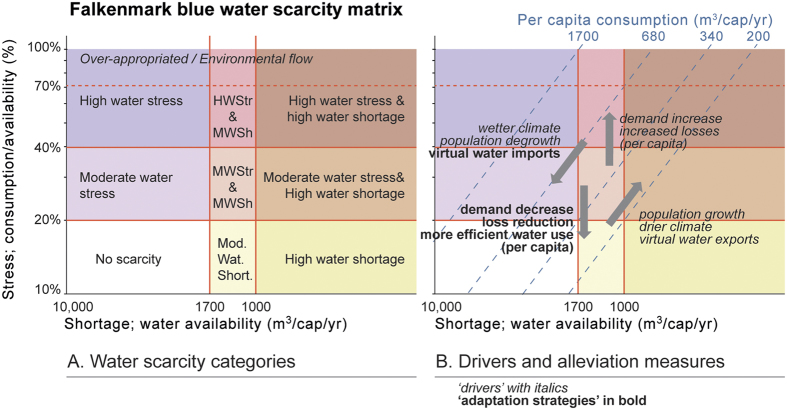
Water scarcity matrix (adapted from Falkenmark^16^ and Falkenmark *et al*.^17^). (**A**) the water scarcity categories; and (**B**) Drivers and alleviation measures. The diagonal lines in tile B refer to per capita consumption isolines. [Adobe Illustrator CS5 –software was used to create the figure; http://www.adobe.com/products/illustrator.html].

**Figure 5:**
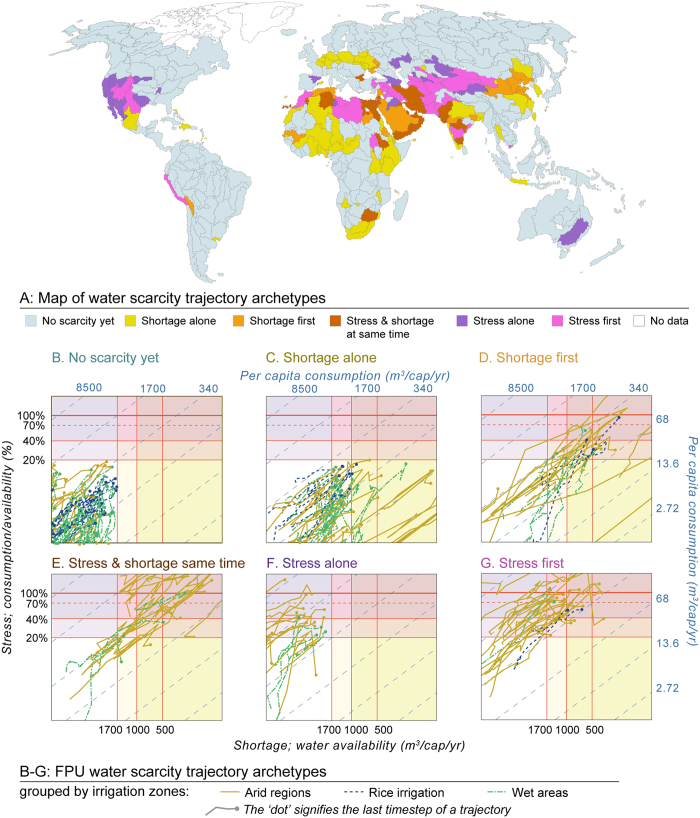
FPU water scarcity trajectories by scarcity archetypes in a map (**A**) and within the Falkenmark matrix (**B–G**). Archetypes categorise FPUs according to their water scarcity status (corresponding to position on the plot) and where both shortage and stress occur, according to which occurs first (which is related to the level of per capita consumption). The trajectories are grouped based on irrigation zone^20^ they are located in. See Table 2A for definitions and Supplementary Table 2 for percentage of population in each archetype – irrigation zone combination. Note: only FPUs with more than one million people are included. [Adobe Illustrator CS5 and R studio softwares were used to create the figure; http://www.adobe.com/products/illustrator.html, https://www.rstudio.com].

**Figure 6:**
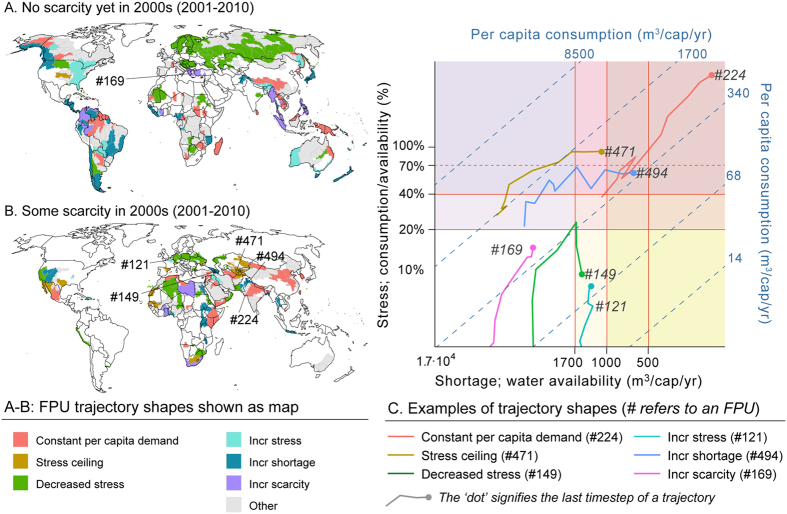
FPU water scarcity trajectory shapes. (**A and B**) FPU shapes shown as map, separated according to whether scarcity has been experienced (**B**) or not (**A**). (**C**) Examples of shapes of FPU water scarcity trajectories. The diagonal lines refer to per capita consumption isolines and numbers to FPUs (location indicated in tile **B**). See Table 2B for definition of each shape category and Supplementary Fig. 2 for each FPU trajectory categorised by their shape. [Adobe Illustrator CS5 and R studio softwares were used to create the figure; http://www.adobe.com/products/illustrator.html, https://www.rstudio.com].

Different FPUs show distinct population dynamics, climate patterns, and developments of water consumption per capita. An FPU’s long-term water scarcity trajectory over time is visualised using the Falkenmark matrix^16^ (Fig. 4) that distinguishes between population-driven water shortage and demand-driven water stress, and highlights the relationship with per capita consumption using superimposed diagonal lines. Drivers and adaptation strategies are strongly dependent on the level and type of water scarcity an FPU is experiencing (Fig. 4B). As defined in Table 2 and discussed below, the notions of *archetypes* and *shapes* help to make sense of these trajectories. The archetype refers to the positioning within the Falkenmark matrix (Fig. 5), whilst shape (Fig. 6) refers to the direction of change over time.

### FPU water scarcity trajectories: archetypes

The concept of water scarcity trajectory *archetypes* captures issues related to water scarcity status and per capita consumption. Trajectory archetypes are thus also useful to identify possible adaptation measures in an FPU. Their definitions are summarised in Table 2A while Fig. 5 maps the regions belonging to each archetype, and displays their trajectories. Each archetype is discussed further below.

The archetypes *stress alone* or *stress first* (before shortage) are experienced if per capita consumption is high (Fig. 5F,G), such that scarcity is demand-driven. FPUs in this category would thus benefit most from demand-side oriented adaptation strategies. The archetypes *shortage alone* or *shortage first* (Fig. 5C,D) are experienced if per capita consumption is low, such that scarcity is population-driven. This calls for supply-side adaptation strategies in particular. This division of adaptation strategies also corresponds to a distinction between ‘soft’ behaviour-change and ‘hard’ infrastructure-based solutions, respectively^1^,^17^,^25^,^26^ (Supplementary Table 1).

Specifically, using a threshold of stress of 20% and a per capita water availability (shortage) threshold of 1700 m^3^ cap^−1^ yr^−1^, the switch-over point between *stress first* and *shortage first* occurs at a per capita consumption of 340 m^3^ cap^−1^ yr^−1^ (Fig. 5A–F; Methods). The *stress and shortage at same time* archetype is a borderline case, in which per capita consumption varies near that switch-over point. For that archetype, both adaptation strategies may be relevant. When an FPU is of a *no scarcity* archetype, no direct adaptation measures are necessary. However, as population grows, the per capita consumption of an FPU sets it on a trajectory towards either stress first or shortage first, and so the above introduced guidelines may apply.

For *stress first* and *stress alone* archetypes, the need for demand management rather than supply side measures^1^ is motivated by the common ideological point of view that high per capita water consumption should be reduced. In practice, however, there seems to be a tendency to meet demand first, for example in the case of trajectories with a constant per capita demand shape (see Fig. 6C). This might be explained in terms of the “hydraulic mission”^27^, common around the world in the 20^th^ century, which aims to dominate nature in order to increase food production and provide water and food security. This has to some extent been curbed by increased emphasis on social and environmental impact assessment^27^,^28^. Ideally, adaptation strategies should focus first on increasing water productivity (domestic, agricultural, and industrial) or on shifting to lower water footprint goods and services. The latter might include reducing virtual water exports^29^ and/or increasing virtual water imports^30^. Several of these actions would not be captured by the data and analysis applied, and may have already occurred, as suggested by recent studies^29^,^31^,^32^,^33^,^34^.

For cases where shortage occurs before stress, supply-side options are in principle preferred because lower per capita water consumption provides less potential for demand-side intervention than when stress occurs first. There are, however, two main ways to handle water shortage: (i) increasing available water, or (ii) limiting population. Available water can be increased by using desalination (in coastal areas)^35^, introducing physical water transfers^36^,^37^ and/or reducing non-productive evaporation^38^. Increased storage capacity is likely to play a smaller role at decadal scale, but is a common strategy to increase seasonal or inter-annual water availability. Emigration and lowered birth rates may limit population, but are perhaps better treated as side-effects of other developments rather than explicit water scarcity strategies. Moreover, an area can adapt to water shortage by using the strategies to reduce per capita water consumption. Possibilities for reducing water requirements include more efficient irrigation^39^, reduction of food losses^40^, reduction of water-intensive goods^41^,^42^, and reduction of leakages in public supply systems^43^.

The potential for reducing blue water consumption notably depends on green water availability (soil water from precipitation), especially in the case of agriculture^13^, but also, for example, on urban parks and golf courses. Areas with reliable green water resources tend to have lower blue water consumption, and hence less stress. While this study does not quantify green water availability, it does show that different archetypes occur depending on the reason for irrigation consumption (which is the largest water-consumption sector in most areas). As discussed in Siebert *et al*.^20^, irrigation is notably driven by: (i) the desire to make agriculture possible in arid areas; (ii) the desire to increase productivity in semi-arid and temperate areas; or (iii) weed-suppression by controlling the water level when growing rice. The results by irrigation zones^20^ (see Fig. 5 for trajectories by irrigation zones, and tabulated results for population in Supplementary Table 2) indicate, for example, that most of the high per capita consumption *stress first* (90% of FPUs within those archetypes) or *stress alone* (82% of FPUs) trajectories occur in arid regions, consistent with higher crop water requirements due to irrigation. *Shortage alone* in turn occurs commonly in wet areas (50% of FPUs), consistent with low water requirements and high population pressure.

In practice, it appears that shortage is not directly tackled until stress occurs. Moderate shortage is tolerated, perhaps buffered by low consumption and other water sources, such as virtual water imports, green water and fossil groundwater. This avoids tackling the underlying issue of population growth, and stress is reached some time later. For example, in North-eastern Mainland China, some FPUs have experienced shortage since before 1905, and others more recently since 1925 and 1975 (Supplementary Fig. 3). Stress followed years or decades later, as population grew. Groundwater and a number of inter-basin transfers are already in use, and additional south-north transfers are in development^44^,^45^. These FPUs are good examples where per capita blue water consumption is low enough that shortage occurred first. There is, however, significant potential for further reductions due to large virtual water exports, which could avoid the need for inter-basin transfers^45^.

### FPU water scarcity trajectories: shapes

When FPU trajectories are distinguished by their *shape*, it is possible to understand the dynamics of consumption over time, and how that has impacted on the scarcity type (shapes are summarised in Table 2B; example trajectories for each shape are shown in Fig. 6C and all trajectories in Supplementary Fig. 2). Further, shapes can be used to assess what needs to be done for an FPU to be put on a sustainable pathway, avoiding both water stress and water shortage in the long term. The majority of FPUs show significant temporal variation in per capita water consumption, stress, and shortage, consistent with the expected tension between population growth, water supply and demand management. In general, achieving sustainable water consumption on a decadal scale requires a combination of stabilising population, enforcing limits of sustainable supply, mitigating impacts of water stress and/or reducing water requirements.

All these strategies are likely to be required to deal with FPUs in the shape categories *increasing scarcity* and *other*. The former face both incessant population growth and intensification of water consumption, which currently leads to strictly increasing stress and shortage (6.6% of global population in 2000s, Fig. 6), for example in parts of the Balkans (FPU 169, Fig. 6). The *other* shape category (32.2% of the population) shows complex trajectories for which specific recommendations cannot be made without other economic or demographic data.

In FPUs where the trajectory shape is determined by *constant per capita demand* (29% of population), changes in scarcity are predominantly determined by population growth. *Constant per capita demand* is visible as a (relatively) straight diagonal trajectory in the Falkenmark matrix (Figs 4B and 6C). As long as per capita consumption is kept in check, stabilising population is an effective strategy for FPUs with any trajectory shape as it avoids increases in shortage and total consumption, and hence stress.

In FPUs with strictly *increasing stress but varying shortage* (4.9% of population), consistent intensification of water consumption is the key concern, for example in northern France (FPU 121, Fig. 6). Recognising the socio-economic importance of exploitation of the local water resource and potential difficulty in curbing water consumption, achieving sustainability may involve mitigation measures to allow greater water consumption than would otherwise be possible. Examples include improving water allocation and other governance mechanisms, providing storage and channelling engineering works, optimising environmental flows, and benefit-sharing to compensate other impacted users. This corresponds to the idea of ‘decoupling’ growth from impacts^46^.

In FPUs with strictly *increasing shortage but varying stress* (15% of population), the key concern is strong population growth, as in northern India (FPU 494, Fig. 6). Recognising that addressing the drivers of population growth may take time, achieving sustainability may involve reducing local water requirements, so that consumption does not grow in parallel with population. This corresponds to decoupling growth from resource use and may be achieved by improved water productivity or decreasing water-dependent production^40^,^41^. Decoupling from resource use already appears to be occurring in many areas, as shown by decreasing trends for per capita consumption (Fig. 1). In FPUs where irrigation is important, per capita consumption is particularly influenced by area equipped for irrigation and a combination of irrigation efficiency and climate effects. However, the most prominent examples of decoupling from local resource use are FPUs dominated by cities, taking as an example FPU 307 in western Africa (32 million people in 2000s), which includes the megacity of Lagos in Nigeria. While some food and other water-dependent products are produced in the hinterland, they can also be imported from elsewhere (along with virtual water)^47^. Such areas can therefore have relatively low *local* blue water requirements, mainly for domestic and industrial water supply (83% of total water consumption at FPU 307). The sustainability of such FPUs depends largely on their interactions with regional and global water resources.

In addition to cases where trends suggest that decoupling is occurring, the analysis identifies some cases with a *stress decrease*-shape (10% of population), or where stress stabilised (*stress ceiling*-shape, 2% of population). In most cases, this occurs as a result of decreases in consumption, but appears to be driven often by socio-economic factors rather than limited water availability. Results show that FPUs that have reached a stress ceiling are mostly those with high per capita consumption that suffer water stress alone (*cf.* Figs 3 and 6B) in North America, Central Asia, or Africa. However, *stress ceilings* occur even with a stress level of 10% (e.g. in Northern Africa), and *decreases in stress* in FPUs that are not water scarce in large parts of the former Soviet Union (Fig. 6A), following the dissolution of the Soviet Union. This may thus be related to the region’s political and economic changes. Consistent with the idea of a “hydraulic mission”^27^,^28^, dams and canals increased supply to allow irrigation demand to expand. Reductions in consumption then occurred not just due to improvements in irrigation efficiency but also due to a shift from exported cotton (and virtual water^29^) to food self-sufficiency in the newly independent nation states^48^,^49^. Water scarcity trajectories and their sustainability are closely tied with other socio-economic and political issues.

## Discussion

This study highlights key issues in understanding global historical water scarcity and pathways for future adaptation. Considering both forms of water scarcity, this analysis provides an improved understanding of blue water consumption and trajectories of past water scarcity development globally at sub-national level for the entire 20^th^ century. The results show that more people are under water scarcity than previously estimated (Supplementary Table 4).

Only a few previous studies assessed historical water scarcity using multiple water use sectors^10^,^19^,^50^, and even then only for the past 50 years. This study’s results compare well with previous trends and estimates of water consumption since 1960, the starting period of existing assessments^10^,^50^ (Supplementary Table 3). The largest improvement in this study, in terms of water consumption trends, is the use of historical spatially explicit irrigation maps^20^ rather than national values. This results in large differences in the location and extent of irrigation areas, particularly in large countries, such as the USA^20^.

Findings for population under stress and shortage separately also show good agreement with existing studies of historical water scarcity (Supplementary Table 4). The existing studies focus on water stress alone^10^ or water shortage alone^7^, or assess both forms of scarcity at only one or two time steps^16^ or scenarios^29^, with the exception of one study^18^ that assesses the interannual variability of blue water scarcity. Assessing both shortage and stress over several decades provides additional insights on the development of water scarcity. The FPU-level trajectories show signs not just of differences in resource endowments and local history, but also similarities due to shared problems and diffusion of solutions, suggestive of a global shared destiny for which collaboration is essential. Classifying sub-national water scarcity trajectories in terms of *archetypes* (Fig. 5) helps to highlight possible adaptation actions to cope with shortage and/or stress, depending on the level of water consumption in per capita terms. Classifying trajectories in terms of their *shape* (Fig. 6) helps to highlight different approaches to put FPUs on a sustainable pathway. Nearly all FPUs show an increase in scarcity over time as population increases (Fig. 6; Supplementary Fig. 2), indicating that understanding of scarcity adaptation actions and pathways to sustainability will only become more important in the future. These historical trajectories provide a common foundation from which further work can dig deeper to identify mistakes to avoid repeating, and past successes worth replicating, in order to better tackle future challenges of water scarcity.

As noted in Introduction, results presented correspond to a well-defined scope focussed on scarcity associated with a long-term view of consumptive blue water use. The selected indicators are widely adopted and can be linked to previous studies^8^,^9^,^10^,^14^,^18^. Additional information sources that would allow more sophisticated water scarcity analysis are not available for the entire study period. These include water quality, technological and social access to water and trade of virtual water. Future studies could include these aspects.

Furthermore, the analysis is commensurate with the significant uncertainty involved in the datasets and models used to cover the globe for the past 110 years^51^,^52^. In this study, two important datasets are combined: water availability and water use, both provided by the WaterGAP2 model. In order to reduce uncertainty in water availability estimates, the model has been calibrated in a basin-specific manner against mean annual river discharge using 1319 gauging stations^53^. Previous studies have reported that the model performs well in relation to other global hydrological models when compared to observations^51^, giving confidence in our water availability estimates. Water use data, on the other hand, is viewed as particularly uncertain^54^. For example, in a multi-model comparison, Wada *et al*.^55^ show that modelled irrigation demand compares reasonably well to country-scale reported values (deviations in the range of +/− 15% in most cases) and conclude that most models are capable of simulating regional variability in irrigation water demand across the globe. Since irrigation constitutes the largest share to global total water consumption and is the dominant water-consuming sector in many parts of the world, it is very likely to also dominate the uncertainty in estimated total water consumption.

We compared the water consumption data of this study to two previous studies assessing the past water consumption^10^,^50^ (Supplementary Table 3), and found that the consumption estimates vary on the order of 35%, this study being the most conservative one. When our water scarcity results were compared to existing studies^10^,^18^ (Supplementary Table S4), we found that estimates of global population under shortage, and population under stress vary on the order of 15% and 30% respectively.

Besides these two key input data products, various assumptions have been made in the analysis itself. A notable assumption relates to the thresholds used to differentiate different states of water stress and shortage. Whilst these assumed thresholds directly affect the amount of population living under water scarcity, they do not affect the trajectory lines in the Falkenmark matrix themselves. Correspondingly, the shapes of the trajectories are not affected by these thresholds. However, trajectory archetypes would somewhat be impacted, as changing these thresholds would mean a specific FPU reaches a certain level of scarcity a decade earlier or later.

As a result, our emphasis is on drawing coherent insights rather than providing precise estimates. In this context, specific numbers represent one possible realisation in the context of significant uncertainty. This is important when comparing our results for a specific year with other studies. The key conclusions of this study are, however, robust, namely the interpretation of sub-national shortage and stress trajectories and the importance of population growth and per capita water consumption in determining local development of scarcity. They are consistent with existing understanding, and strongly influenced by patterns in input data (e.g. population growth and expansion of irrigation area) that are independent of other assumptions made in the analyses.

The analytical approach used and the initial insights it provides could also be used as a foundation for further research. Additional information about uncertainty could be obtained by systematically repeating the analysis with other models and forcing datasets, as has been done in comparable contexts^5^. This would, however, require a carefully chosen, meaningful set of scenarios. A range of different assumptions can be used regarding scarcity thresholds and indicators, focussing on different issues delimiting different perspectives on safe and just operating spaces for socio-ecological systems^3^,^56^. Calculating indicators at seasonal^11^,^57^ or annual scale^18^,^58^ would allow investigation of how shortage and stress occur at shorter time scales, more closely related to every-day operations rather than long-term planning. Ideally, availability would be tied to access, which would help alleviate problems related to selection of spatial scale^59^. Focussing on water quality^60^,^61^, unsustainable water sources^62^, and on spatially explicit environmental flow requirements^4^,^63^ (the thresholds used for water stress assume global environmental flow requirements of 30%^17^) would explicitly identify the portion of available water that should not be used to avoid stress according to different criteria. Similarly, focussing on self-sufficiency of water and food^12^,^58^,^64^ would identify specific water requirements for shortage, though it would also require greater consideration of both blue and green water^13^.

Whether self-sufficiency is required is particularly relevant in the context of trade^65^ and virtual water transfers^31^, which are not captured in this study. From an economics perspective, scarcity is not intrinsically problematic, but rather raises questions of optimal allocation of the scarce resources, trade to make use of comparative advantages, and the inclusion of externalities. Prominent issues include the role of water quality and safety^66^, and accessibility and equity determined by social, economic and political circumstances^25^,^67^,^68^,^69^,^70^,^71^. Linking the trajectories to other datasets may help deepen understanding, expanding and better explaining the shapes introduced here (Table 2B), and how they relate to historical and future drivers as well as limits to adaptation.

## Methods

### Analysis unit: Food production units

**Figure 7:**
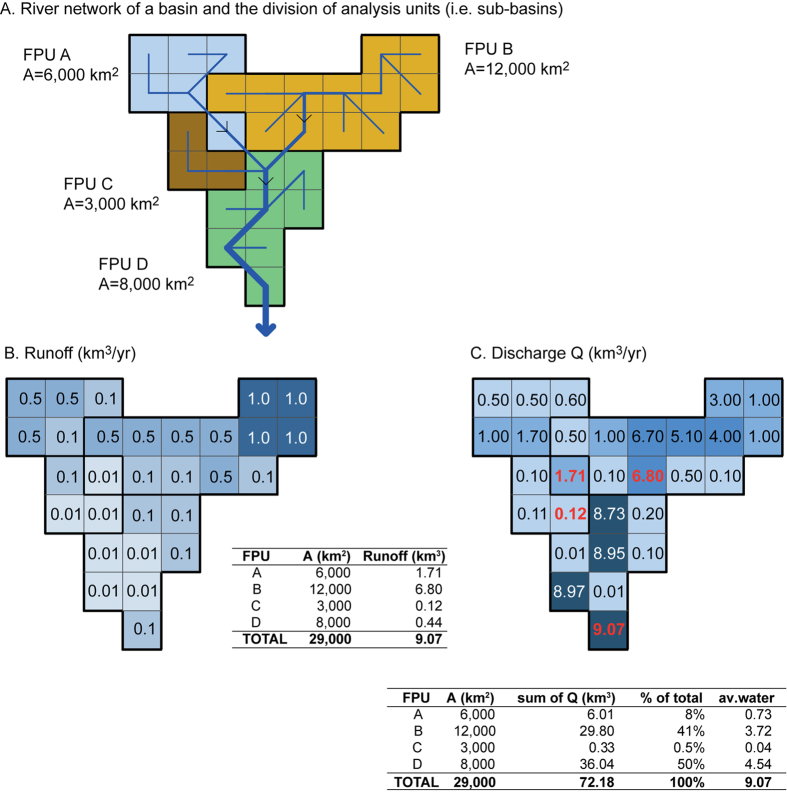
Water availability calculations in a large basin with several FPUs, i.e. each FPU is a sub-basin for the large basin. A: schematic illustration of a basin with four FPUs; B: Runoff of each grid cell in km^3^ yr^−1^; and C: discharge of each grid cell in km^3^ yr^−1^. The share of available water resources is calculated as the sum of discharges of each grid cell within an SBA divided by the sum of discharges of all grid cells within a basin. The available water resources are then calculated by multiplying that share with the total available runoff of the whole basin. [Adobe Illustrator CS5 –software was used to create the figure; http://www.adobe.com/products/illustrator.html]

This study used food production units (FPUs), a combination of river basin and administrative boundaries^7^,^72^,^73^, as an analysis unit. These are reported to be suitable for water scarcity studies^7^,^58^. For this project, a set of FPUs were developed that are consistent with the basin delineation of the WaterGAP2 hydrological and water use models, resulting in 548 FPUs. It is important to use the same delineation for FPUs as watersheds of the WaterGAP2 model, as the way water availability is dealt with (see Fig. 7) requires that FPUs do not cross the borders of large river basins. Results are also aggregated from the FPU scale to regional (*n* = 12) scale. The regions are based on UN macro regions aggregating the countries to larger units^74^ with the difference that some of the largest regions were divided into smaller regions by Kummu *et al*.^7^ to be more suitable for (historical) water analyses.

### Water availability

This analysis used the global hydrological model WaterGAP2^53^ to derive gridded estimates for runoff and river discharge at 30 arc-min spatial resolution for the study period of 1901–2010. Based on daily meteorological forcing fields and spatially distributed physiographic information (e.g. soil, land cover), the model simulates the terrestrial water cycle by a sequence of storage equations for the storage compartments canopy, snowpack, soil, renewable groundwater, and surface water bodies. For this study, simulations were driven by WATCH Forcing Data (WFD) which is available for the period 1901–2001^75^. Since it is not recommended to combine WFD with other similar data-sets^53^,^76^ in order to derive full coverage over the study period 1901–2010, simulations for the period beyond the year 2001 were based on 1990s climate forcing.

Since this analysis focuses on long-term trends in water scarcity, the 10-yr annual average over each decade was calculated for both discharge and runoff to compensate for inter-annual variability. These data were then used to assess the water availability in each FPU. The calculation of water availability can be divided into two cases:

- In cases when an FPU consisted of one basin or several small basins, water availability was simply the sum of annual runoff generated within the area of a specific FPU.
- In cases of large river basins that were divided into several FPUs, a simple ‘water sharing rule’ was used to assign the available freshwater resources within each FPU^5^,^12^. This was developed in a way that it would be usable for both water shortage and water stress calculations, i.e. the sum of water availability of the FPUs within the basin cannot exceed the annual runoff of the basin. The water sharing rule was based on a discharge proportion of FPUs within a basin multiplied with the annual runoff, as illustrated in Fig. 7.

### Water consumption

The water use model of WaterGAP2 simulates water withdrawals and consumption of the following sectors: i) irrigation, ii) livestock farming, iii) thermal electricity production, iv) manufacturing industries, and v) households and small businesses (domestic).

To indicate the area equipped for irrigation (AEI), the analysis used the HID product by Siebert *et al*.^20^, which gives spatially explicit AEI for the entire 20^th^ century. The proportion of irrigated harvested rice area was based on the MIRCA-2000 dataset^77^. The proportions were kept at year 2000 level throughout the study period due to lack of historical data. As in the case of the water availability simulations (see above), to simulate the irrigation water consumption beyond 2001, climate forcing data from the 1990s were used. The estimate of consumption for the 2000s should therefore not be included when assessing trend in per capita consumption. Irrigation water consumption is the amount of water that must be applied to the crops by irrigation in order to achieve optimal crop growth. Monthly consumptive irrigation requirements are therefore based on climate, the spatial extent of AEI and crop type (rice and non-rice). Return flows, i.e. water withdrawal minus water consumption, which account for water that infiltrates and returns to the water cycle, are not quantified in this study.

Livestock water consumption was calculated on the basis of gridded information on the number of livestock units and water consumption per head and year, taking into account 10 livestock types^21^. Due to limited data prior to the year 1960, livestock water consumption for the period of 1900–1960 was kept at the level of 1960. Overall, this may lead to an underestimation or overestimation in livestock water consumption depending on the FPU^78^, which is expected to be minor as the amount of livestock water consumption is small compared to the other sectors. Water consumption estimates for electricity, manufacturing, and domestic sectors were based on the methodologies described in Flörke *et al*.^19^. In brief, domestic water consumption is estimated from population and domestic water use intensity, taking into account structural and technological changes. Country-scale water consumption in the manufacturing sector is calculated from manufacturing structural water use intensity, gross value added, and consumption coefficients; again taking into account technological change. The amount of water withdrawn and consumed for cooling purposes in thermoelectric power production is determined from the annual thermal electricity production and the water use intensity of each power station, distinguishing three cooling system types (once-through, pond, and tower cooling systems) and several fuel types (fossil/biomass/waste-fuelled, nuclear, natural gas/oil combined, coal/petroleum residuum-fuelled). Based on this information, the model approach distinguishes 14 combinations of plant type (PT) and cooling system (CS). In 2010, about 2.8% of cooling water abstractions evaporated, i.e. most of the water withdrawn was discharged back into rivers (Flörke *et al*.^19^).

To get the total water consumption, all the water use sectors are summed together. Trends in per capita consumption (see background in Fig. 1A) were determined with the Mann-Kendall test, calculating the Kendall correlation of demand with time. A *p*-value of 0.05 was used as part of a two-sided test of whether the correlation was statistically significantly different from zero.

### Water stress calculations

The indicator of blue water stress is the water use to availability ratio. We use consumption rather than withdrawals, such that water ‘use’ means that water is no longer available for other users. The indicator was calculated for each decade and for each FPU. The water stress thresholds used are, however, those for the withdrawal-based water stress index (WSI) developed by Falkenmark^16^, and used by a number of other studies^8^,^10^,^57^,^78^:

1. WSI <0.2: no water stress
2. WSI = 0.2–0.4: moderate water stress
3. WSI >0.4: high water stress

Using withdrawals risks over-estimating the actual stress as a substantial part of the withdrawals are available for downstream users as return flows^22^,^23^. On the other hand, using water consumption, as in this study, might underestimate the water stress. Recent work by Munia *et al*.^79^ uses consumption and withdrawals as minimum and maximum levels of scarcity, respectively. They show that the difference between these two estimates results in an 18 percent point difference in the amount of population under water stress. Similar uncertainties in the absolute amount of people under water scarcity should be considered for the numbers quoted in this study. This may also be worthwhile approach for future work. Finally, it should be stressed that the thresholds used assume a global environmental flow requirements of 30%^17^.

### Water shortage calculations

For water shortage calculations the analysis is based the water crowding index (WCI) developed by Falkenmark^17^,^80^. WCI is calculated by dividing the water availability by total population of an FPU. Here, historical, spatially explicit, population data is from HYDE 3.1^81^. The water shortage thresholds are as follows:

1. WCI >1700 m^3^ cap^−1^ yr^−1^: no water shortage
2. WCI = 1000–1700 m^3^ cap^−1^ yr^−1^: moderate water shortage
3. WCI <1000 m^3^ cap^−1^ yr^−1^: high water shortage

### Water scarcity matrix and related calculations

To illustrate the combination of water stress and water shortage, the analysis used the Falkenmark water scarcity matrix (Fig. 4). By plotting water stress against shortage over time, water scarcity trajectories were derived for each FPU. These trajectories in turn were categorised for archetypes and shapes (Table 2, and see below).

The formulas used for the indicators mean that for any combination of stress and shortage, per capita consumption can also be calculated (see diagonal lines in Fig. 4B). For example, consider the point where an FPU is classified as under both water stress and water shortage:


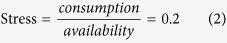



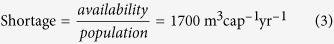


The corresponding per capita consumption can be calculated for those values of stress and shortage (see also Fig. 4B):


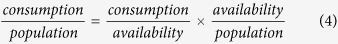



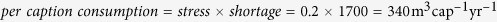


For a given per capita consumption, this formula can be rearranged to identify whether an FPU would already be stressed when the shortage threshold is reached (shortage = 1700 m^3^ cap^−1^ yr^−1^).


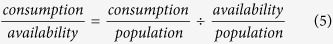



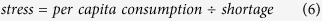



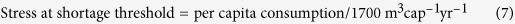


Therefore, the following interpretation can be made when assuming shortage of 1700 m^3^ cap^−1^ yr^−1^:

If per capita consumption = 340 m^3^ cap^−1^ yr^−1^ → stress = 0.2 (stress and shortage same time)

If per capita consumption >340 m^3^ cap^−1^ yr^−1^ → stress >0.2 (stress occurs first)

If per capita consumption <340 m^3^ cap^−1^ yr^−1^ → stress <0.2 (shortage occurred first)

### Scarcity archetypes

The scarcity archetypes define the water scarcity status and level of per capita consumption (see Table 2A). Scarcity categorisation for archetypes is based on the lowest stress (20%) and shortage thresholds (1700 m^3^ cap^−1^ yr^−1^). ‘No scarcity yet’ are FPUs that have never reached the lowest threshold of water stress (20%) or shortage (1700 m^3^ cap^−1^ yr^−1^). For ‘Shortage alone’, water availability has passed the threshold of 1700 m^3^ cap^−1^ yr^−1^, but stress has remained below the threshold of 20%. ‘Stress alone’ occurs where stress exceeds 20% but water availability (i.e. shortage) has never dropped below 1700 m^3^ cap^−1^ yr^−1^. ‘Stress first’, ‘Shortage first’ and ‘Stress and shortage at same time’ occur when the trajectory has exceeded both the stress and shortage thresholds, sub-categorised according to which type of strategy is reached first.

### Scarcity shapes

The scarcity shapes, in turn, divide the trajectories into categories based on their shape when plotted in the Falkenmark matrix. Specific rules for each shape were developed as outlined in Table 2B.

## Additional Information

**How to cite this article**: Kummu, M. *et al*. The world’s road to water scarcity: shortage and stress in the 20th century and pathways towards sustainability. *Sci. Rep.* **6**, 38495; doi: 10.1038/srep38495 (2016).

**Publisher's note:** Springer Nature remains neutral with regard to jurisdictional claims in published maps and institutional affiliations.

## Supplementary Material

Supplementary Information

Supplementary Dataset 1

Supplementary Dataset 2

## Figures and Tables

**Table 1:** Global population (in millions) under different kinds of water scarcity during the 20^th^ century.

Water stress [-]	Water shortage [m^3^ cap^−1^ yr^−1^]	Description	‘Population in millions (% of total)					
			1900s	1920s	1940s	1960s	1980s	2000s
		Global population	1711	1996	2418	3366	4869	6512
0.2–0.4	>1700	M WStr	45 (2.6%)	75 (3.8%)	58 (2.4%)	81 (2.4%)	59 (1.2%)	104 (1.6%)
>0.4	>1700	H WStr	3 (0.2%)	9 (0.4%)	49 (2.0%)	59 (1.7%)	72 (1.5%)	19 (0.3%)
<0.2	1000–1700	M WSh	48 (2.8%)	117 (5.9%)	207 (8.6%)	262 (7.8%)	871 (18%)	1569 (24.1%)
<0.2	<1000	H WSh	77 (4.5%)	58 (2.9%)	10 (0.4%)	58 (1.7%)	99 (2.0%)	468 (7.2%)
0.2–0.4	1000–1700	M WStr + M WSh	5 (0.3%)	4 (0.2%)	7 (0.3%)	33 (1.0%)	32 (0.7%)	204 (3.1%)
>0.4	1000–1700	H WStr + M WSh	31 (1.8%)	23 (1.1%)	26 (1.1%)	38 (1.1%)	192 (3.9%)	103 (1.6%)
0.2–0.4	<1000	M WStr + H WSh	0 (0.0%)	36 (1.8%)	96 (4.0%)	59 (1.7%)	249 (5.1%)	191 (2.9%)
>0.4	<1000	H WStr + H WSh	29 (1.7%)	51 (2.6%)	80 (3.3%)	231 (6.9%)	477 (9.8%)	1133 (17.4%)
>0.2	or <1700	TOTAL	238 (13.9%)	373 (18.7%)	533 (22.1%)	822 (24.4%)	2053 (42%)	3791 (58.2%)

M WStr refers to moderate water stress, H WStr to high water stress, M WSh to moderate water shortage and H WSh to high water shortage. See matrix of the scarcity classes in Fig. 4A.

**Table 2:** Description of the trajectory categorisation used in the study: A) scarcity trajectory archetypes (see Fig. 5); and B) shape of trajectory (see Fig. 6).

Type of trajectory*	Description
*A. Archetype*	
No scarcity yet	Per capita available water >1700 m^3^ cap^−1^ yr^−1^ and stress <0.2 always, corresponds to FPU trajectories confined to bottom-left of Falkenmark matrix
Shortage alone	Per capita available water <1700 m^3^ cap^−1^ yr^−1^ for some decades and stress <0.2 always, corresponds to FPU trajectories confined to bottom of Falkenmark matrix
Stress alone	Stress >0.2 for some decades, but per capita available water <1700 m^3^ cap^−1^ yr^−1^ always, corresponds to FPU trajectories confined to left of Falkenmark matrix
Shortage first	Per capita available water >1700 is reached before stress >0.2, includes FPUs that have reached top-right of Falkenmark matrix, and generally where per capita consumption is low (<340 m^3^ cap^−1^ yr^−1^)
Stress first	Stress >0.2 is reached before per capita available water >1700 m^3^ cap^−1^ yr^−1^, includes FPUs that have reached top-right of Falkenmark matrix, and generally where per capita consumption is high (>340 m^3^ cap^−1^ yr^−1^)
Stress and shortage at same time	Stress >0.2 and per capita available water >1700 m^3^ cap^−1^ yr^−1^ both reached in the same decade. Includes FPUs that have reached top-right of Falkenmark matrix and where per capita consumption is either close to 340 m^3^ cap^−1^ yr^−1^, highly variable or the FPU has always been subject to water scarcity within the data period studied
*B. Shape of trajectory*+	
Increasing scarcity	Both stress and shortage increase every decade
Increasing shortage	Shortage increases every decade, stress may vary
Increasing stress	Stress increases every decade, shortage may vary
Decreased stress	(Max stress – stress in 2005)/(max stress – min stress) > 0.2 Final stress is less than 20% of its maximum
Stress ceiling	|Stress_d_- Stress in 2005|<0.04 for some 1915 ≤ d ≤ 1995 and |Stress_d_- Stress in 2005|<0.06 from some 1915 ≤ d ≤ 1995 onward i.e. stress becomes close to its final value, and stays close to its final value from some decades, but in both cases not from the start, and not just before the end
Constant per capita demand	Linear fit to stress=m.(1/shortage)+c has R^2^>0.95

^*^Trajectories are characterised in two different ways.

^+^Trajectories are assigned to the first applicable category.
