# Supplementary material for: The world’s road to water scarcity: shortage and stress in the 20th century and pathways towards sustainability
Source: Sci Rep. 2016 Dec 9;6:38495. doi: 10.1038/srep38495 (PMC5146931; doi:10.1038/srep38495)
Supplement: Supplementary Information [file srep38495-s1.pdf]

Supplementary information

The world's road to water scarcity: shortage and stress in the 20th century and pathways towards sustainability

Kummu M[1*#], Guillaume JHA[1,2*], de Moel H[3], Eisner S[5], Flörke M[5], Porkka M[1], Siebert S[6], Veldkamp TIE[3], Ward PJ[3]

1. Water & Development Research Group (WDRG), Aalto University, Espoo, Finland

2. National Centre for Groundwater Research and Training & Integrated Catchment Assessment and Management centre, The Fenner School of Environment and Society, The Australian National University, Australia

3. Institute for Environmental Studies (IVM), Vrije Universiteit Amsterdam, Amsterdam, Netherlands

5. Center for Environmental Systems Research (CESR), University of Kassel, Germany

6. Institute of Crop Science and Resource Conservation (INRES), University of Bonn, Germany

[*] contributed equally to work
[#] correspondence to matti.kummu@aalto.fi

Supplementary tables

Supplementary Table 1. Hard infrastructure-based & soft behaviour-change alleviation measures[1, 2, 3, 4]*.*

Hard	Soft
Increasing storage capacity (reservoirs, dams)<br>Desalination of sea water<br>Water transfer<br>Ground water use<br>Expansion of rain-water storage<br>Virtual water imports	Resource allocation<br>Improve water productivity/efficiency via pricing or subsidies:<br>• domestic: water-based sanitation, consumption patterns<br>• industry: water use, recycling ratios, withdrawals<br>• agriculture: irrigation technology, crop characteristics, crop calendar, area planted

Supplementary Table 2. Percentage of population, a) in FPUs split by archetype, b) in FPUs split by irrigation zone

	a) by archetype				b) by irrigation zone			
Archetype	All FPUs	Arid regions	Rice irrigation	Wet areas	Arid regions	Rice irrigation	Wet areas	total
No scarcity yet	41%	11%	55%	51%	7%	43%	50%	100%
Same time	8%	19%	NA	6%	68%	NA	32%	100%
Shortage alone	31%	16%	36%	37%	14%	38%	49%	100%
Shortage first	11%	23%	8%	5%	56%	25%	19%	100%
Stress alone	2%	4%	NA	2%	61%	NA	39%	100%
Stress first	7%	27%	1%	0%	95%	5%	0%	100%
Total	100%	100%	100%	100%				

Supplementary figures

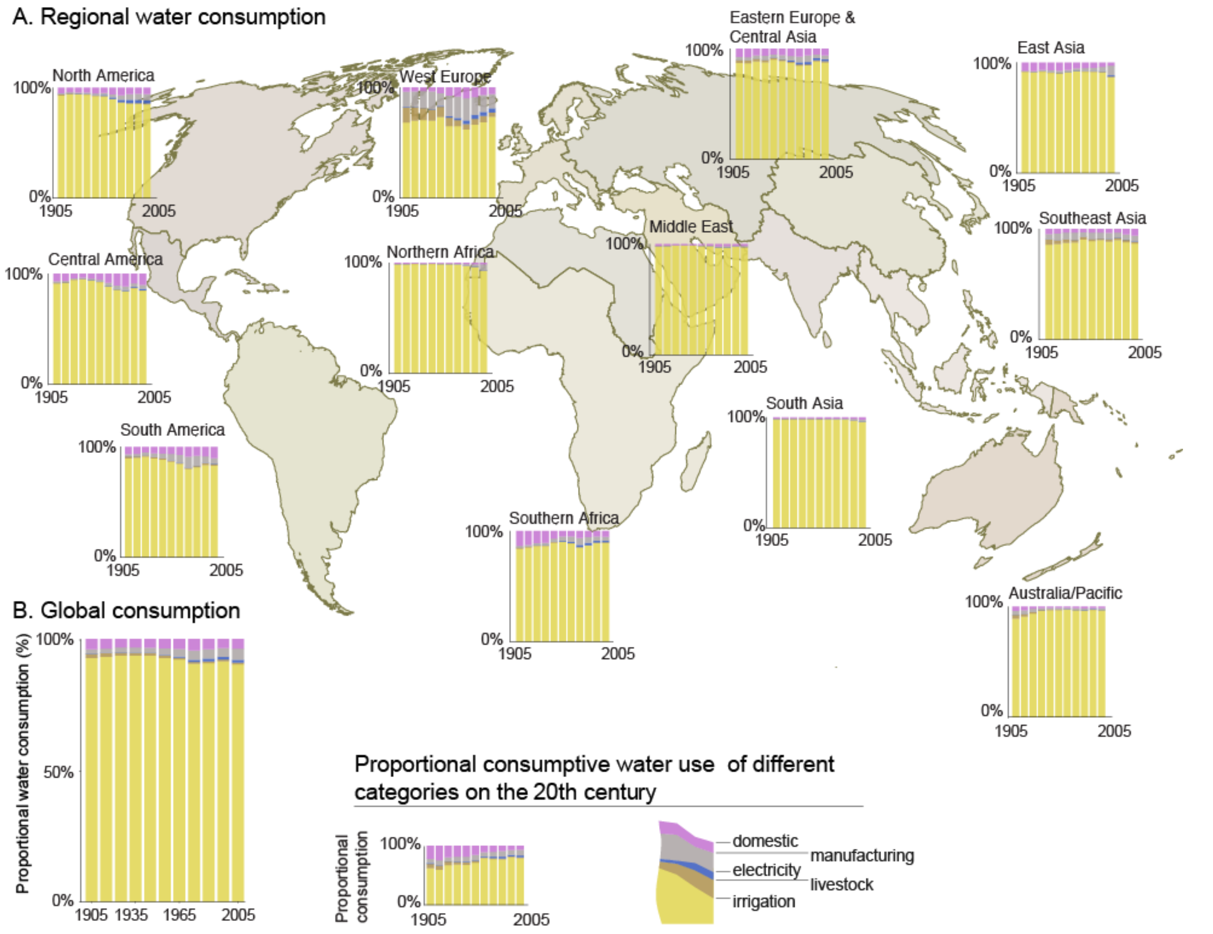

Supplementary Figure 1. Proportional consumptive water use by different categories for each region (A) and for the entire globe (B). [Adobe Illustrator CS5 and ArcGIS 9.2 softwares were used to create the figure; http://www.adobe.com/products/illustrator.html, http://www.esri.com]

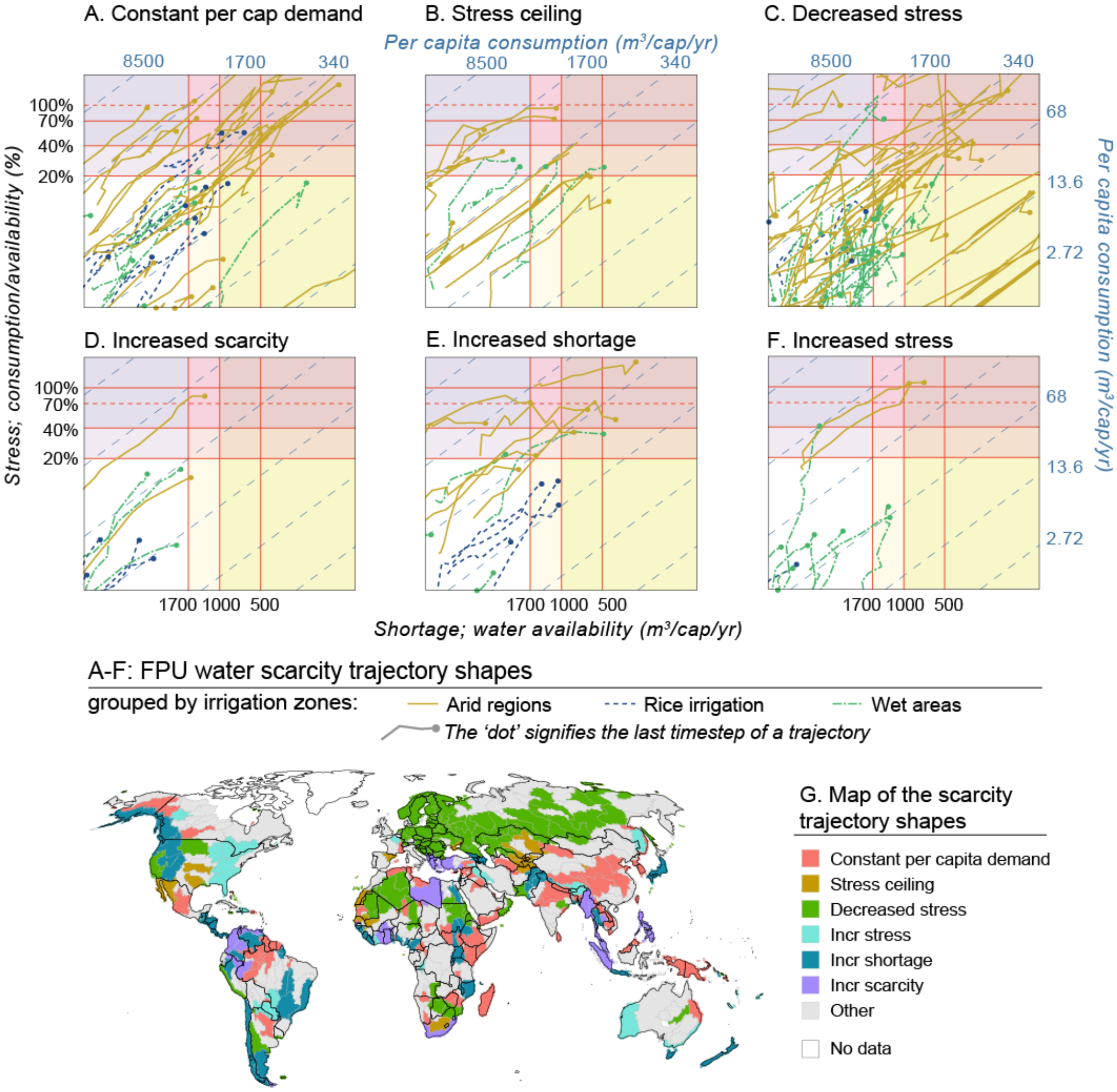

Supplementary Figure 2. Trajectory shapes. A-F: trajectories separated by shape of trajectory (note: not all FPUs shown due to the axes limits); G: map of the trajectory shapes. See Table 2B in the main text for the definitions. [Adobe Illustrator CS5 and R studio softwares were used to create the figure; http://www.adobe.com/products/illustrator.html, https://www.rstudio.com]

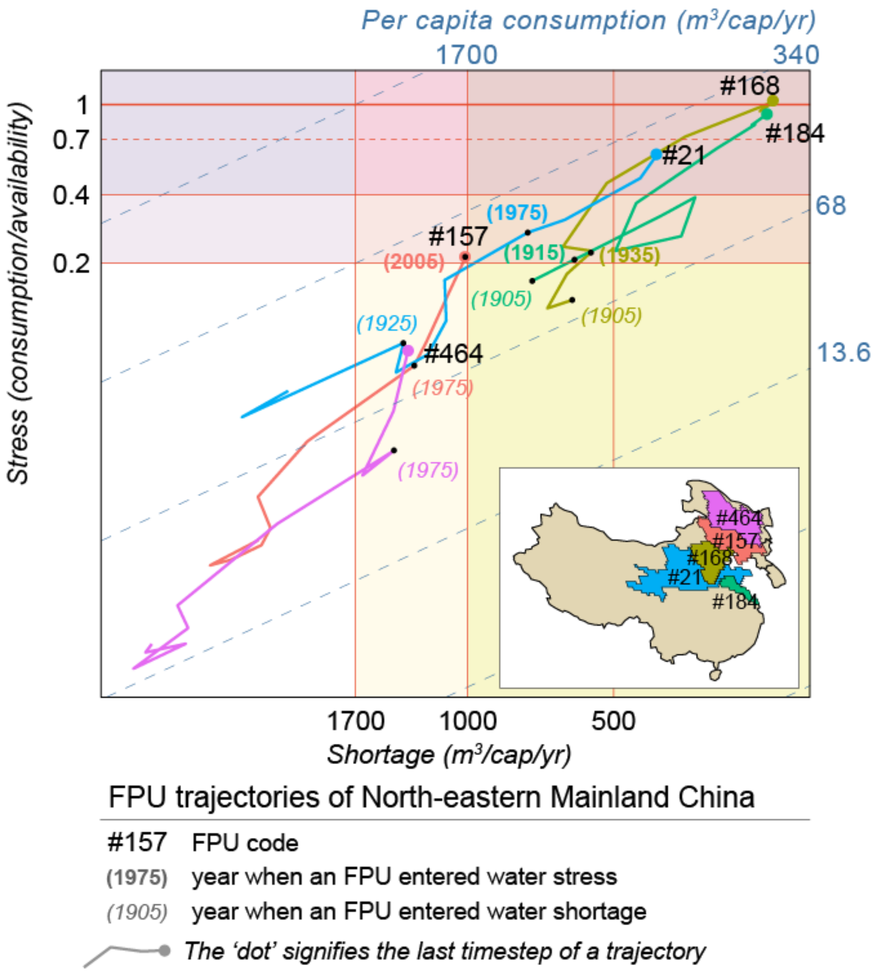

Supplementary Figure 3. Scarcity trajectories for selected FPUs in north-eastern Mainland China. Labels in brackets show first year in which shortage (italics) and stress **(bold)** *are first experienced. [Adobe Illustrator CS5 and R studio softwares were used to create the figure; http://www.adobe.com/products/illustrator.html, https://www.rstudio.com]*

Comparison of water consumption and scarcity results with existing studies

The current global water consumption results agree rather well with the existing estimates (Supplementary Table 3). With both compared datasets[5, 6], however, the results obtained are higher at the beginning of the comparison period and smaller in the year 2000. The results for the period 1960-1990 are within ±100 km^3 yr^{-1} when compared to those of Wada et al[6], but are 315 km^3 yr^{-1} lower in the year 2000. The difference with Shiklomanov[5] is within ±50 km^3 yr^{-1} for period 1900-1940, but the gap gradually increases from 240 in year 1960 to 666 km^3 yr^{-1} in year 2000 (Supplementary Table 3). Existing studies report that the per capita water consumption continued to increase or stabilised since the 1980s, while simulation results of this study indicate that it is currently uncertain whether per capita water consumption is showing a long-term downward trend, or simply a temporary decrease (Figure 1). In other words, the current model runs are unable to determine whether the rate of population growth has exceeded water consumption growth (decreasing per capita water consumption) or vice versa.

Supplementary Table 3. Comparison of our water consumption results to the existing studies of Shiklomanov[5] and Wada et al[6].

Water consumption [km^3 yr^{-1}]	1900	1940	1960	1970	1980	1990	2000
Shiklomanov	331	617	1086	1341	1686	1982	2182
Wada et al	-	-	818	976	1275	1456	1831
This study [a]	358	577	860	1018	1198	1349	1500

[a] in case of this study, the values are average values for the decade starting in the given year (i.e. year 1980 is average over 1981-1990).

The present results indicate that since the 1950s-1960s, the population under water scarcity increased very rapidly, both in absolute terms and relative to total population. This is in line with the findings of existing literature[6, 7, 8]. The findings of this study differ in estimated population under scarcity as both dimensions of water scarcity were included to the current assessment. While previous studies found that 17-19% (10-12%) of the population was under water shortage[7, 8](water stress[6, 8]) in 1960 and 45-50%(28-34%) in 2000, current results indicate that total population under water scarcity (shortage and/or stress) was somewhat larger (24% and 58%, respectively; see Supplementary Table 4). However, when assessing the results separately for water stress and water shortage, they agree rather well with the existing literature[6, 7] (Supplementary Table 4). It should be noted that some of the differences might be explained by slightly different reference years. As we use ten-year average figures, the results represent the years 1965 and 1985, which we compared to the years 1960 and 1980 in the literature. There are also some methodological differences, such as analysis unit and model selection, which might also explain part of the disagreement.

Supplementary Table 4. Comparison of our water scarcity results to the existing studies, in case of population and percentage of global population under water shortage or water stress.

	Year	Water shortage		Water stress		Total [a]
		Moderate (1000-1700 m^3 cap^{-1} yr^{-1})	*High (<1000 m^3 cap^{-1} yr^{-1})*	*Moderate (0.2 – 0.4)*	*High (>0.4)*	
Kummu et al [7]	1960	287 (10%)	284 (9%)			571 (19%)
	1980	969 (22%)	710 (16%)			1679 (38%)
	2005	951 (15%)	2296 (35%)			3247 (50%)
Wada et al [6]	1960			100 (3%)	200 (7%)	300 (10%)
	1980			300 (7%)	500 (11%)	800 (18%)
	2000			500 (8%)	1200 (20%)	1700 (28%)
Veldkamp et al [8] [b]	1960	473 (17%)		326 (12%)		551 (20%)
	2000	2550 (45%)		1900 (34%)		2830 (50%)
This study	1965	333 (10%)	348 (10%)	173 (5%)	328 (10%)	822 (24%)
	1985	1095 (22%)	825 (17%)	340 (7%)	741 (15%)	2053 (42%)
	2005	1876 (29%)	1792 (28%)	499 (8%)	1255 (19%)	3791 (58%)

[a] in case of Kummu et al [7] total population under water shortage, Wada et al (2011) total population under water stress, and this study total population under a water scarcity, including both, water shortage and water stress.

[b] Veldkamp et al [8] report only combined values for moderate and high water shortage and moderate and high water stress.

References

1. Falkenmark M. Growing water scarcity in agriculture: future challenge to global water security. *Philosophical Transactions of the Royal Society A: Mathematical, Physical and Engineering Sciences* **371**, (2013).

2. Falkenmark M, Berntell A, Jägerskog A, Lundqvist J, Matz M, Tropp H. On the Verge of a New Water Scarcity: A Call for Good Governance and Human Ingenuity. In: *SIWI Policy Brief*. Stockholm International Water Institute (SIWI) (2007).

3. Gleick PH. Water in crisis: Paths to sustainable water use. *Ecol Appl* **8**, 571-579 (1998).

4. Gleick PH. Global Freshwater Resources: Soft-Path Solutions for the 21st Century. *Science* **302**, 1524-1528 (2003).

5. Shiklomanov IA. Appraisal and assessment of world water resources. *Water International* **25**, 11-32 (2000).

6. Wada Y, van Beek LPH, Bierkens MFP. Modelling global water stress of the recent past: on the relative importance of trends in water demand and climate variability. *Hydrology and Earth System Sciences* **15**, 3785-3808 (2011).

7. Kummu M, Ward PJ, de Moel H, Varis O. Is physical water scarcity a new phenomenon? Global assessment of water shortage over the last two millennia. *Environmental Research Letters* **5**, 034006 (2010).

8. Veldkamp TIE*, et al.* Changing mechanism of global water scarcity events: impacts of socioeconomic changes and inter-annual hydro-climatic variability. *Global Environmental Change* **32**, 18-29 (2015).
