# Supplementary material for: The world’s road to water scarcity: shortage and stress in the 20th century and pathways towards sustainability
Source: Sci Rep. 2016 Dec 9;6:38495. doi: 10.1038/srep38495 (PMC5146931; doi:10.1038/srep38495)
Supplement: Supplementary Dataset 2 [file srep38495-s3.zip › read_me.rtf]

Supplementary dataset 2: spatial delineation of FPUs for Kummu & al: “The world’s road to water scarcity: shortage and stress in the 20th century and pathways towards sustainability”This spatial dataset ‘kummual_road_to_scarcity_Supplementary_dataset_2_FPU.tif’ provides a spatial reference to used food production units (FPUs). It can be opened with QGIS, ArcGIS or any other GIS software. It can be also imported to RStudio or Matlab.  Fileformat: GeoTIFFResolution: 30 arc-minProjection: WGS84Extent: Lat: S90° to N90°; Lon: W180° to E180°For more information: matti.kummu@aalto.fi 
