# Supplementary figures and images for: The world’s road to water scarcity: shortage and stress in the 20th century and pathways towards sustainability
Source: Sci Rep. 2016 Dec 9;6:38495. doi: 10.1038/srep38495 (PMC5146931; doi:10.1038/srep38495)

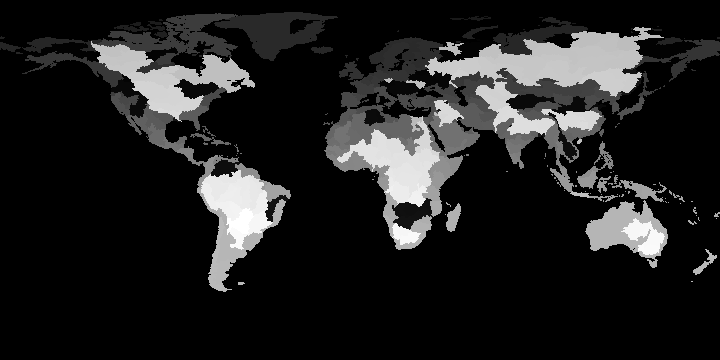

Supplement: Supplementary Dataset 2 [file srep38495-s3.zip › kummual_road_to_scarcity_Supplementary_dataset_2_FPU.tif]
